# Association of colchicine use for acute gout with clinical outcomes in acute decompensated heart failure

**DOI:** 10.1002/clc.23830

**Published:** 2022-04-28

**Authors:** Mary E. Roth, Melissa E. Chinn, Steven P. Dunn, Kenneth C. Bilchick, Sula Mazimba

**Affiliations:** ^1^ Department of Pharmacy University of Virginia Health Charlottesville Virginia USA; ^2^ Department of Medicine—Cardiovascular Medicine University of Virginia Health Charlottesville Virginia USA; ^3^ Present address: Melissa E. Chinn Yale New Havel Hospital, 20 York Street New Haven 06510 CT USA

**Keywords:** colchicine, gout, heart failure, in‐hospital mortality

## Abstract

**Background:**

Gout is a common comorbidity in heart failure (HF) patients and is frequently associated with acute exacerbations during treatment for decompensated HF. Although colchicine is often used to manage acute gout in HF patients, its impact on clinical outcomes when used during acute decompensated HF is unknown.

**Methods:**

This was a single center, retrospective study of hospitalized patients treated for an acute HF exacerbation with and without acute gout flare between March 2011 and December 2020. We assessed clinical outcomes in patients treated with colchicine for a gout flare compared to those who did not experience a gout flare or receive colchicine. The primary outcome was in‐hospital all‐cause mortality.

**Results:**

Among 1047 patient encounters for acute HF during the study period, there were 237 encounters (22.7%) where the patient also received colchicine for acute gout during admission. In‐hospital all‐cause mortality was significantly reduced in the colchicine group compared with the control group (2.1% vs. 6.5%, *p* = .009). The colchicine group had increased length of stay (9.93 vs. 7.96 days, *p* < .001) but no significant difference in 30‐day readmissions (21.5% vs. 19.5%, *p* = .495). In a Cox proportional hazards model adjusted for age, inpatient colchicine use was associated with improved survival to discharge (hazards ratio [HR] 0.163, 95% confidence interval [CI] 0.051−0.525, *p* = .002) and a reduced rate of in‐hospital CV mortality (HR 0.184, 95% CI 0.044−0.770, *p* = .021).

**Conclusion:**

Among patients with a HF exacerbation, treatment with colchicine for a gout flare was associated with significantly lower in‐hospital mortality compared with those not treated for acute gout.

## 1 INTRODUCTION

Gout is a common comorbidity in heart failure (HF) patients and is the result of monosodium urate crystal deposition in joints and periarticular tissues.^1^ Gout is associated with significant morbidity, mortality, and healthcare costs.^1^, ^2^, ^3^ Diuretics are known to precipitate hyperuricemia and increase the risk of gout flares through mechanisms related to decreased uric acid secretion and increased uric acid reabsorption.^4^, ^5^ Studies have estimated the prevalence of gout in HF patients to be approximately 16%−40%, and one study found that 56% of hospitalized HF patients had hyperuricemia.^6^, ^7^, ^8^, ^9^

The therapeutic agents commonly used for an acute gout flare include colchicine, steroids, and nonsteroidal anti‐inflammatory drugs (NSAIDs). However, steroids and NSAIDs are often avoided in HF because of the legitimate concerns of fluid retention and HF exacerbation.^10^ In addition to its role in gout, colchicine's anti‐inflammatory effects are also highly beneficial in the treatment and prevention of other cardiac conditions such as pericarditis.^11^ Colchicine has also recently shown broader cardiovascular (CV) outcomes benefit in high‐risk patients, particularly those with coronary artery disease (CAD) or history of myocardial infarction (MI). However, the impact of colchicine use during gout flares on outcomes in patients with acutely decompensated HF is unknown. The purpose of this study was to assess clinical outcomes in patients treated for an acute HF exacerbation and receiving colchicine for an acute gout flare.

## 2 METHODS

### 2.1 Study design

This single center, retrospective cohort study compared clinical outcomes in those receiving colchicine for the treatment of an acute gout flare versus those without a gout flare among patients with an acute HF exacerbation at an academic medical center. Adult patients (age ≥ 18 years) admitted between March 2011 and February 2020 with an acute HF exacerbation who received initial intravenous (IV) diuretics were eligible for inclusion. Patients were identified using ICD 9 and ICD 10 codes for acute HF exacerbation. Patients treated with colchicine during the admission for a documented acute gout flare were included in the treatment group, while those not given colchicine during the admission were presumed not to have had a gout flare and were included in the control group. Patients were excluded if they had end‐stage renal disease on hemodialysis, any history of transplantation or underwent transplantation during the admission, and any history of left ventricular assist device (LVAD) or LVAD implantation during admission. Patients receiving colchicine for indications other than acute gout or admitted for reasons other than acute decompensated HF were also excluded. All patients were included for analysis on an intention‐to‐treat basis. The study was approved by the Institutional Review Board before data collection. Study covariates were collected from the electronic medical record and data warehouse of medical records.

### 2.2 Outcomes

The primary outcome was in‐hospital all‐cause mortality. Secondary outcomes included hospital length of stay (LOS), 30‐day readmissions, and time to death. This study also compared the primary and secondary outcomes between patients with a prior history of gout, a first diagnosis of gout, and the control group. A post hoc analysis was completed to evaluate in‐hospital CV mortality and time to CV death. Baseline characteristics included age, gender, and ejection fraction (on the most recent imaging including echocardiogram, nuclear stress test, or angiography), comorbidities, and home HF and gout medications.

### 2.3 Statistical analysis

The primary outcome of in‐hospital all‐cause mortality and secondary outcome of 30‐day readmissions were compared using the Pearson *χ* ^2^ test, while LOS was assessed using the Mann−Whitney *U* test. Reverse Kaplan−Meier curves stratified by colchicine treatment were constructed to evaluate the difference in survival. Time to death and time to CV death during the hospital admission were assessed using bivariable Cox proportional hazards regression with censoring at 4 weeks. The proportional hazards assumption in this case was easily verified by inspection of the Kaplan−Meier survival curves. Other baseline demographics and secondary outcomes compared categorical variables with Pearson *χ* ^2^ tests, while continuous variables were compared using unpaired two‐sample *t*‐tests/analysis of variance (ANOVA) and the Mann−Whitney *U*/Kruskal−Wallis tests. Multivariable logistic regression was performed to assess the association of in‐hospital colchicine with survival to discharge with adjustment for key covariates. Multivariable Cox proportional regression was used to assess differences in time to death with adjustment for age. The *α* value for all statistical tests was set at .05. R and SPSS statistical software were used in the analysis.

## 3 RESULTS

### 3.1 Baseline characteristics

**Figure 1:**
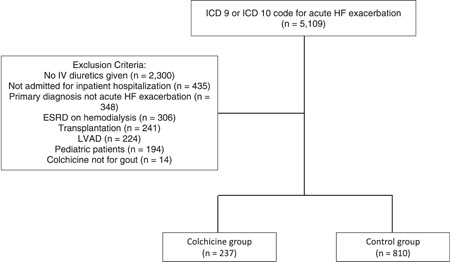
CONSORT flow diagram. The CONSORT flow diagram is shown for the cohort. ESRD, end stage renal disease; HF, heart failure; IV, intravenous; LVAD, left ventricular assist device

A total of 5109 patient encounters had an ICD 9 or ICD 10 code for acute HF exacerbation during the study period (Figure 1). The final cohort included 1047 patient encounters after exclusions as indicated in Figure 1 and was then stratified into colchicine and control groups as determined a priori. Baseline characteristics were similar between groups, with the exception of age, sex, and comorbidities of gout and alcohol use (Table 1). Patients in the colchicine group were more likely to be male and younger compared to the control group. A majority of the patients had HF with reduced ejection fraction (54.1%), and there was no significant difference in HF classifications between groups. Patients who received colchicine for an acute gout flare were more likely to have a history of gout (50.2% vs. 3.2%, *p* < .001) and alcohol use (3.4% vs. 1.2%, *p* = .026) compared with the control group. Admission serum creatinine was significantly higher in the colchicine group than the control group (1.73 vs. 1.44 mg/dl, *p* < .001). There was no significant difference in the change from admission serum creatinine to discharge serum creatinine between groups. Among the patient encounters with a uric acid level checked during the admission, uric acid was significantly higher in the colchicine group compared with the control group (10.63 vs. 8.26 mg/dl, *p* = .002). There was no significant difference in admission B‐type natriuretic peptide level between groups.

**Table 1:** Baseline Characteristics^a^

	Colchicine	Control	Total	*p* Value
	(*N* = 237)	(*N* = 810)	(*N* = 1047)	
Age, years	66.6 ± 12.8	69.4 ± 13.9	68.8 ± 13.7	.005
Male sex, *n* (%)	180 (75.9)	469 (57.9)	649 (62)	<.001
HF type, *n* (%)				.461
HFrEF (EF ≤ 40%)	136 (57.4)	428 (53.2)	564 (54.1)	
HFmrEF (EF 41−49%)	23 (9.7)	95 (11.8)	118 (11.3)	
HFpEF (EF ≥ 50%)	78 (32.9)	282 (35)	360 (34.5)	
Admission SCr, mg/dl	1.73 ± 0.95	1.44 ± 0.78	1.51 ± 0.81	<.001
Admission BNP, pg/ml^b^	1272 ± 1316	1319 ± 1474	1308 ± 1440	.698
Uric acid, mg/dl^c^	10.63 ± 3.68	8.26 ± 3.52	9.97 ± 3.77	.002
Comorbidities, *n* (%)				
Gout	119 (50.2)	26 (3.2)	145 (13.8)	<.001
CAD	141 (59.5)	470 (58.0)	611 (58.4)	.687
Hypertension	94 (39.7)	281 (34.7)	375 (35.8)	.160
Diabetes mellitus	80 (33.8)	261 (32.2)	341 (32.6)	.658
Hyperlipidemia	41 (17.3)	109 (13.5)	150 (14.3)	.137
Alcohol use	8 (3.4)	10 (1.2)	18 (1.7)	.026
Tobacco use	10 (4.2)	27 (3.3)	37 (3.5)	.516
Home Medications, *n* (%)				
Allopurinol	65 (27.4)	75 (9.3)	140 (13.4)	<.001
Colchicine	97 (40.9)	22 (2.7)	119 (11.4)	<.001
Febuxostat	5 (2.1)	5 (0.6)	10 (0.9)	.053
Probenecid				
ACEi/ARB/ARNi	82 (34.6)	332 (40.9)	414 (39.5)	.077
Beta‐blocker	173 (72.9)	459 (56.7)	632 (60.4)	<.001
MRA	45 (19.9)	90 (11.1)	135 (12.9)	.001
Hydralazine	24 (10.1)	33 (4.1)	57 (5.4)	<.001
Nitrate	35 (14.8)	73 (9)	108 (10.3)	.011
Diuretic				.001
Furosemide	104 (43.9)	377 (46.5)	481 (45.9)	
Bumetanide	62 (26.2)	70 (8.6)	132 (12.6)	
Torsemide	12 (5.1)	13 (1.6)	25 (2.4)	
None	59 (24.9)	350 (43.2)	409 (39.1)	
Thiazide	52 (21.9)	109 (13.5)	161 (15.4)	.001
Inotrope	4 (1.7)	5 (0.6)	9 (0.9)	.124
Digoxin	30 (12.7)	49 (6.1)	79 (7.5)	.001
Non‐DHP CCB	7 (2.9)	23 (2.8)	30 (2.9)	.926

Abbreviations: ACEi, angiotensin converting enzyme inhibitor; ARB, angiotensin receptor blocker; ARNi, angiotensin receptor neprilysin inhibitor; BNP, B‐type natriuretic peptide; CAD, coronary artery disease; HFmrEF, heart failure with mid‐range ejection fraction; HFpEF, heart failure with preserved ejection fraction; HFrEF, heart failure with reduced ejection fraction; MRA, mineralocorticoid receptor antagonist; Non‐DHP CCB, nondihydropyridine calcium channel blocker; SCr, serum creatinine.

^a^ Plus−minus values are means ± SD.

^b^ Admission BNP was only available for 846 patients (189 patients in the colchicine group and 657 patients in the control group).

^c^ Uric acid was only available for 98 patients (71 patients in the colchicine group and 27 patients in the control group). If there were multiple uric acid levels during admission, the first level was recorded.

### 3.2 Primary outcome analysis

A total of 58 patients (5.5%) died during admission, five in the colchicine group and 53 in the control group (2.1% vs. 6.5%, *p* = .009), that is, a lower in‐hospital all‐cause mortality in the colchicine group (Table 2). A subgroup analysis was conducted to assess outcomes with the colchicine group stratified based on prior documented history of gout versus de novo gout presentation. In‐hospital all‐cause mortality was not significantly different between patients with a new gout diagnosis compared to those with a prior history of gout (3.4% vs. 0.8%, *p* = .213).

**Table 2:** Primary and secondary outcomes

	Colchicine	Control	Total	*p* Value
	*N* = 237	*N* = 810	*N* = 1047	
Primary outcome				
In‐hospital mortality, *n* (%)	5 (2.1)	53 (6.5)	58 (5.5)	.009
Secondary outcomes				
30‐Day readmissions, *n* (%)	51 (21.5)	158 (19.5)	209 (20)	.495
Hospital LOS (days), mean±​​​​​​ ​SD	9.93 ± 8.10	7.96 ± 8.42	8.4 ± 8.38	<.001

Abbreviations: LOS, length of stay; SD, standard deviation.

### 3.3 Secondary outcomes analysis

The 30‐day readmissions were not significantly different between the colchicine and control groups (21.5% vs. 19.5%, *p* = .495) (Table 2). In the subgroup analysis, 30‐day readmissions remained similar when comparing patients with de novo gout to those with a prior history of gout (22.9% vs. 20.2%, *p* = .611). Mean LOS was significantly increased in the colchicine group compared to the control group (9.93 vs. 7.96 days, *p* < .001) (Table 2). In the subgroup analysis, LOS was significantly increased in the de novo presentation of gout group compared to the control group (10.52 vs. 7.96 days, *p* < .001) and in the prior history of gout group compared to the control group (9.35 vs. 7.96 days, *p* = .006). There was no significant difference in LOS between those with a first diagnosis of gout and those with a prior history of gout (*p* = .272). In a post hoc analysis, in‐hospital CV mortality censored at 4 weeks was significantly lower in the colchicine group than the control group (0.89% vs. 3.93%, *p* = .02).

### 3.4 Stratified Kaplan−Meier analysis and Cox proportional hazards regression during hospital admission

**Figure 2:**
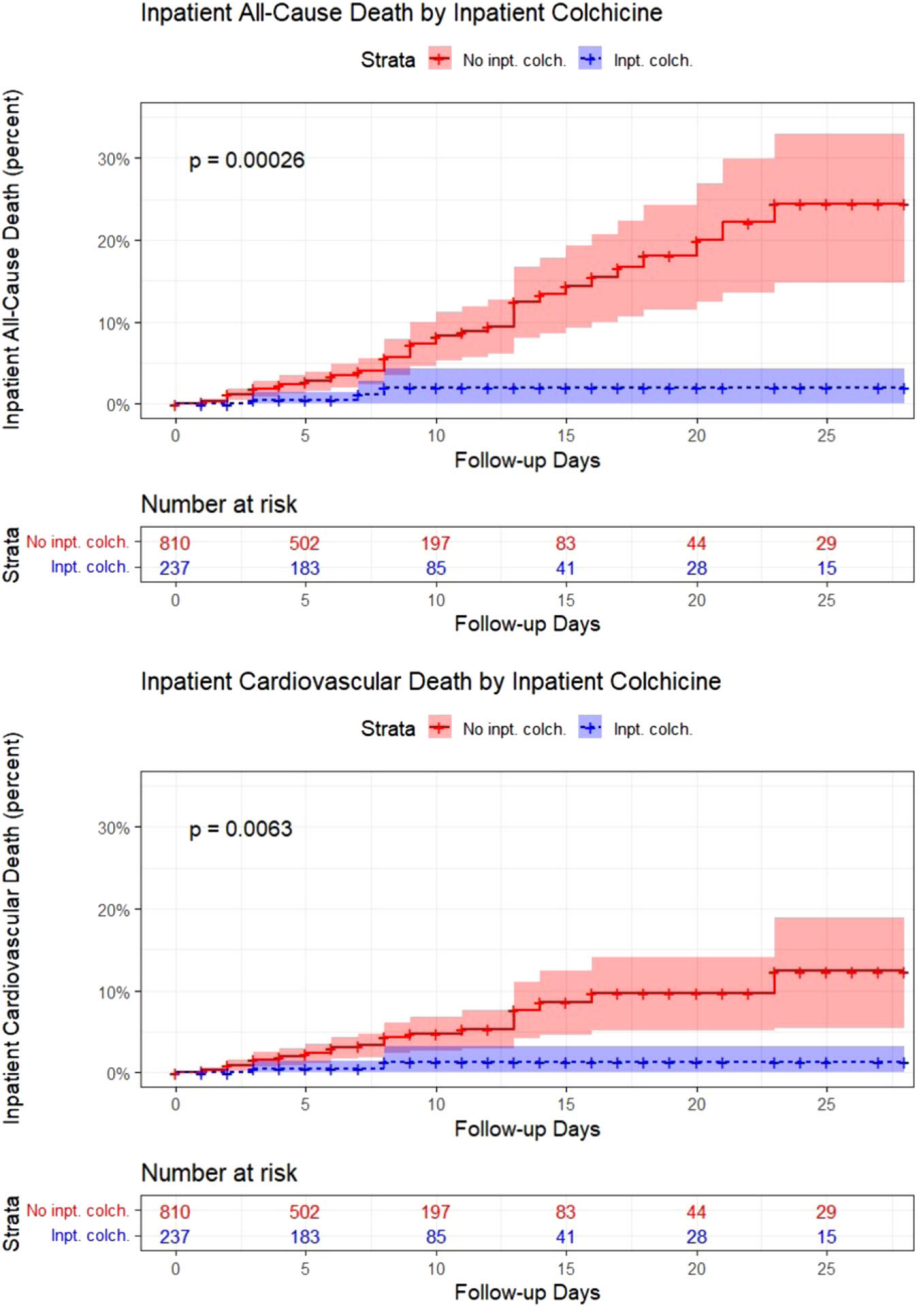
Inpatient all‐cause and cardiovascular (CV) death by inpatient colchicine use. Reverse Kaplan−Meier curves for inpatient all‐cause death and inpatient CV death stratified on inpatient colchicine use are shown (*p* = .00026 and *p* = .0063, respectively)

**Figure 3:**
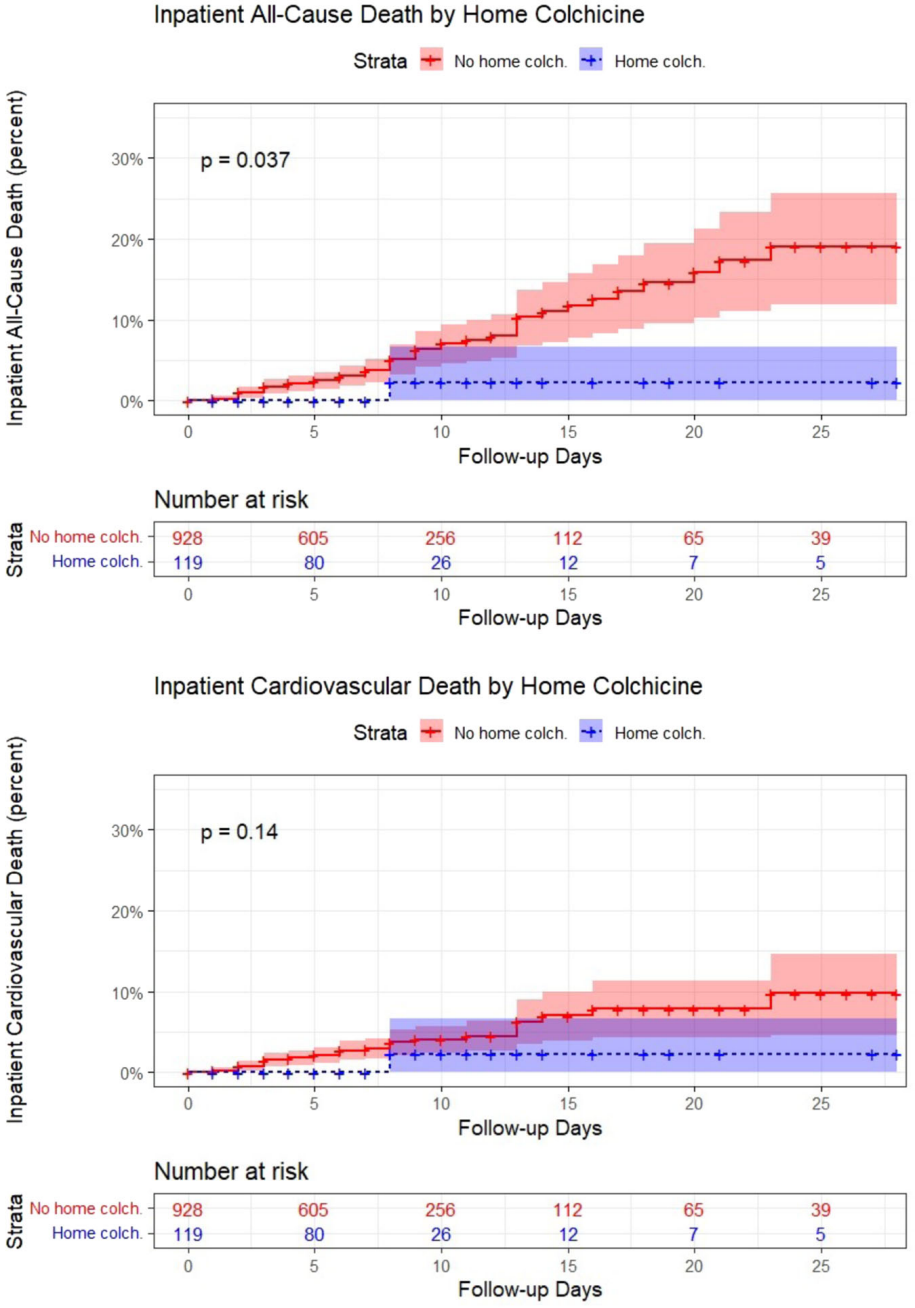
Inpatient all‐cause and cardiovascular (CV) death by home colchicine use. Reverse Kaplan−Meier curves for inpatient all‐cause death and inpatient CV death stratified on home colchicine use are shown (*p* = .037 and *p* = .14, respectively)

Reverse Kaplan−Meier curves stratified by in‐hospital colchicine and censored at 4 weeks are shown in Figure 2. Inpatient colchicine was associated with reduced rates of both in‐hospital all‐cause mortality (log rank *p* = .00026) and in‐hospital CV mortality (log rank *p* = .0063) compared with the control group. In a Cox proportional hazards model adjusted for age, in‐hospital colchicine use was associated with improved survival to discharge (hazard ratio [HR] 0.163, 95% confidence interval [CI] 0.051−0.525, *p* = .002) and a decreased rate of in‐hospital CV mortality (HR 0.184, 95% CI 0.044−0.770, *p* = .021). Reverse Kaplan–Meier curves stratified by home colchicine use were also generated (Figure 3). Home colchicine use was associated with a reduced rate of in‐hospital all‐cause mortality (log rank *p* = .037) but no significant difference in the rate of in‐hospital CV mortality (log rank *p* = .14).

### 3.5 Multivariate logistic regression analysis

A multivariate logistic regression model was performed to evaluate associations of other covariates with in‐hospital all‐cause mortality. These covariates included in‐hospital colchicine use, home beta‐blocker use, inotrope use, age, and diabetes mellitus. In‐hospital colchicine use given for a gout flare was significantly associated with reduced in‐hospital all‐cause mortality (OR 0.322, 95% CI 0.105−0.779, *p* = .02) after adjustment for home beta‐blocker use, inotrope use, age, and diabetes mellitus (*p* < .05 for all in the model).

## 4 DISCUSSION

In this retrospective cohort study, we evaluated the use of colchicine for an acute gout flare during hospitalization for acute decompensated HF. We found that colchicine use during acute HF exacerbation was associated with decreased in‐hospital all‐cause mortality and in‐hospital CV mortality, as well as increased hospital LOS. The incidence of acute gout in this study population was 22.7% of all patient encounters. Although the rate of acute gout while receiving IV diuretics during hospitalization for acute HF is not extensively characterized in the literature, a 2017 study of patients treated with IV bumetanide during hospitalization for acute HF found the incidence of acute gout to be 13.6% over the course of the study.^12^ Our findings highlight the relative high prevalence of acute gout during treatment with IV diuretics for HF exacerbation.

Colchicine is a potent anti‐inflammatory and antiproliferative drug that has been used for both acute gout treatment as well as prevention.^10^ Several studies have reported the safety and beneficial outcomes of colchicine in other cardiac conditions. A recent meta‐analysis of patients with a range of CV disease states evaluated the impact of colchicine on a composite CV outcome, which consisted of the primary outcome of each individual trial and included mortality, acute coronary syndrome, MI, cerebrovascular accident, cardiac arrest, or revascularization. The meta‐analysis found that colchicine use was associated with a 56% decrease in the composite CV outcome (*p* = .0004), as well as a nonsignificant trend toward reduction in all‐cause mortality (relative risk 0.50, *p*  = .08).^11^ Several additional retrospective studies have also demonstrated favorable CV outcomes with the use of colchicine.^13^, ^14^

Recently, prospective, randomized, and placebo‐controlled trials have also examined the potential benefit of colchicine in CV patients. The Colchicine Cardiovascular Outcomes Trial (COLCOT) evaluated the use of colchicine within 30 days after MI, and the Low Dose Colchicine 2 Trial (LoDoCo2) evaluated colchicine in patients with stable CAD. In COLCOT and LoDoCo2, colchicine resulted in a significant reduction in the primary outcomes, which were a composite of CV death and other clinical outcomes in both trials.^25^, ^26^ Additionally, a recent systematic review and meta‐analysis assessed the impact of colchicine in patients with CAD in 13 randomized trials, which included a total of 13 125 patients.^15^ The study found that treatment with colchicine significantly reduced the risk of MI as well as stroke or transient ischemic attack when compared to placebo or standard care. However, colchicine was not associated with a significant reduction in all‐cause or CV mortality.

While many of the existing studies have evaluated colchicine use in patients with CAD or prior MI, to our knowledge, only one trial to date has evaluated colchicine's effects in stable HF.^16^ Investigators randomized stable symptomatic HF patients to receive either colchicine 0.5 mg twice daily or placebo for 6 months. The primary end point, which was the proportion of patients achieving at least one‐grade improvement in New York Heart Association (NYHA) functional status classification, was not significantly different between the two groups (*p* = .365). Colchicine use was associated with a significant decrease in measured inflammatory biomarkers including high sensitivity C‐reactive protein and interleukin‐6. There are some key differences notable in the aforementioned study compared with ours. First, investigators excluded patients hospitalized within the previous 3 months, whereas our population was comprised exclusively of patients admitted for an acute HF exacerbation. Second, patients were given colchicine regardless of gout status, whereas in our study, patients who were given colchicine received it due to an acute gout flare. Third, investigators only included patients with left ventricular ejection fraction ≤40%, in contrast to our study which included HF patients regardless of ejection fraction.

Prior studies have also explored the impact of other gout therapies on HF outcomes. Hyperuricemia has been associated with an increased incidence of HF as well as increased mortality among those with HF. Therefore, uric acid lowering therapies have been considered potential medication candidates for improving HF outcomes. Initial studies demonstrated that allopurinol, a xanthine oxidase inhibitor, was associated with improved endothelial function in HF patients.^17^ Subsequently, the Effects of Xanthine Oxidase Inhibition on Hyperuricemic Heart Failure Patients (EXACT‐HF) study randomized patients (with primarily NYHA Class II and III HFrEF and hyperuricemia) to allopurinol (target dose 600 mg daily) versus placebo for 24 weeks.^18^ The primary outcome, a composite clinical end point based on several factors including survival, worsening HF, and patient global assessment, was not significantly different between the allopurinol and placebo groups. While this prior study failed to demonstrate the efficacy of uric acid lowering with allopurinol on HF outcomes, colchicine has an important distinction related to its anti‐inflammatory properties. This anti‐inflammatory effect is what we believe may underlie the positive findings in our study. Additionally, the EXACT‐HF study enrolled patients in the outpatient setting, while this study focused specifically on patients hospitalized with acute decompensated HF.

The mechanistic underpinnings of the potential beneficial effects of colchicine on CV events may involve its anti‐inflammatory properties on the CV system.^19^ It has been postulated that activated neutrophils are present in atherosclerotic plaques and play a key role in the transformation of a stable to an unstable plaque.^19^ Colchicine's anti‐inflammatory effects and inhibition of neutrophil chemotaxis and activation may play a role in stabilizing plaques and preventing MI or ischemic strokes. Hitherto, the potential utility of colchicine in acute decompensated HF has not been considered. Thus, the underlying mechanistic pathways that could explain the potential benefits of colchicine in the HF population are largely unknown but may be multifactorial. It has been well established that an acute HF admission is associated with increased short term mortality as well as other adverse CV events following an index admission.^20^ Accordingly, a worsening HF event has increasingly been recognized as an end point for enrollment in clinical trials.^21^, ^22^, ^23^ In this sense, acutely decompensated HF represents a distinct vulnerable phenotypic state characterized by multiple neurohormonal perturbations and a heightened proinflammatory milieu.^24^ It is tempting to surmise that our findings demonstrating the favorable effects of colchicine on HF mortality could potentially be explained by the modulating influence of the anti‐inflammatory effects of colchicine on this distinct vulnerable phenotypic phase in the HF trajectory. If indeed our findings are validated, then consideration could be made for designing clinical trials that incorporate anti‐inflammatory agents such as colchicine targeting this vulnerable phase of worsening HF.

## 5 LIMITATIONS

There are some limitations to this study that should be acknowledged. Limitations due to the retrospective design include potential for missing data points, inability to assess diuretic doses received during hospitalization, and reliance on ICD 9 and ICD 10 codes for initial diagnosis of acute HF exacerbation. Readmissions at outside hospitals that are not linked with the institution's electronic medical record may not have been identified. Additionally, in this study the intervention group consisted of patients given colchicine for an acute gout flare, while the control group included those with neither a gout flare nor colchicine treatment. At our institution, most HF patients with acute gout are treated with colchicine, so it would not have been possible to assemble a sufficiently powered control group of acute HF patients who had a gout flare without colchicine treatment. It is possible that acute gout could be a surrogate marker for more effective diuresis or renal dysfunction. However, the existing literature demonstrating worsened outcomes associated with gout and hyperuricemia suggests that the presence of gout in the intervention group would skew the results toward the null hypothesis. As this was an observational study, it is possible that there could still be unmeasured confounders even after statistical adjustment.

## 6 CONCLUSION

This study demonstrated that colchicine use for acute gout flare in hospitalized patients with a HF exacerbation was associated with decreased in‐hospital all‐cause mortality and in‐hospital CV mortality compared with the control group. The use of colchicine was also associated with a longer LOS but similar 30‐day readmissions. Additional large multicenter retrospective and prospective randomized studies are needed to more fully understand the association of colchicine use with outcomes in patients undergoing treatment for HF exacerbations and explore its role as a potential treatment option in this population.

## CONFLICTS OF INTEREST

The authors declare no conflicts of interest.

## Data Availability

Data available on reasonable request from the authors.
